# Organizational psychology on the way to 2065: a challenge to scholars

**DOI:** 10.3389/fpsyg.2015.00948

**Published:** 2015-07-07

**Authors:** Richard E. Boyatzis

**Affiliations:** Organizational Behavior, Case Western Reserve UniversityCleveland, OH, USA

**Keywords:** organizational psychology, employee, leadership, interpersonal relationships, scholars

In the 48 years since I started in the field of Organizational Psychology (OP), a lot of interests have shifted or changed. Some are persistent, like leadership. Our fascination and fear with the forces behind change, innovation, and power continue to provoke research. We are less concerned about efficiency and less likely to study ways to drive people harder. Placement is less of a focus of research, in terms of selection and promotion. Employee participation has morphed from T-groups and sensitivity training, quality circles, employee involvement, business process re-engineering, and bottom up processes to engagement and citizenship. We have experienced philosophical shifts like the emergence of positive and spiritual psychology as disruptive innovations in thought. Some will be the phrenology of the future and pass away as ephemeral intellectual fads, and some will be extinction level events that dramatically change our perspectives and concepts of causality.

OP is becoming a leading integrative arena—truly transdisciplinary. This holds great promise for how our field can contribute into many other fields while maintaining rigor and developing our own concepts. Cleeremans (2010) challenged us to consider psychology a “hub” discipline of the fractionated and silo array of psychologies. In this same way, *OP can become an integrative hub for the social sciences*. Our core intent is and will be to help create or maintain a better life for all in our societies, to seek truth, justice, beauty, and love (or at least get tenure).

There does seem to be eight trends emerging which, I believe, will affect the coming 30–50 years. I am not under the illusion that this list is comprehensive. Therefore, I ask the reader to use this list as their own call to intellectual curiosity to “go where no one has gone before” and help us create a desired future of rigorous and helpful insights through research in OP.

One set of trends address the “what” of a possible future OP:

(1) leadership beyond transformational styles (Bass, 1990) to characteristics like emotional and social intelligence in the context of g and personality;
(2) development of people, dyads, teams, organizations, and communities beyond training to more contextual approaches that are integrated with work and life, like coaching (Van Oosten, 2013) which will integrate many forms of diversity of people and the importance of research on retained learning and what promotes it;
(3) interpersonal relationships (how we interact and effect each other, inclusiveness and diversity) beyond LMX (Graen and Uhl-Bien, 1995) like the concept of high quality connections (Dutton and Heaphy, 2003) shared vision (Clayton, 2014; Miller, 2014) and shared caring through compassion (Boyatzis et al., 2012); and
(4) using organizations to create a greater good and noble purpose in search of social justice, equality, intergroup relations, and well-being, like the work of Laszlo et al. (2014) in flourishing and sustainability, developing true measures of human resource and development profit and loss statements and balance sheets (not merely the financial rendering of them).

The other set of trends address the “how” of a possible future OP:

(5) holistic models and measures, using hormonal systems, neural networks, and nutrition as well as psychological and behavioral variables on effectiveness, mental health, and individual sustainability (Kahneman, 2011; Jack et al., 2012; Boyatzis et al., 2014; Waldman et al., 2014; Passarelli, 2015).
(6) complexity (i.e., non-linear, discontinuous, and emergent) beyond statistical manipulation to normalize the data but direct measures and analysis through mathematical modeling (Coen, 2013) and focusing on interpersonal dynamics (Hazy and Backström, 2013); and
(7) technological, social media, and disruptive innovations, like MOOCs (Massive On-Line Open Courses), social networks, and virtual reality as the basis for collecting research about these phenomena as well as the others mentioned above and experimenting with new pedagogies and assessment of learning and development. This trend could have a useful impact on new pedagogy that might enhance learning (see trend #2 above).
(8) more appropriate and sensitive measurement of variables beyond single source studies to truly multi-method, multi-trait designs using data from many sources like physiological *and* behavioral assessment. For example, discovery that a 5 or 7 point response set in surveys does not work reliably for European samples for whom all social measurement is on a 10 point scale (Batista-Foguet et al., 2014) is the kind of discovery that can change our assessments to be more contextually sensitive.

It is an exciting time of discovery and holds much promise for helping scholars find ways to share their findings and thoughts and create new knowledge.

## Conflict of interest statement

The author declares that the research was conducted in the absence of any commercial or financial relationships that could be construed as a potential conflict of interest.
